# Controlling the Formation of Conductive Pathways in Memristive Devices

**DOI:** 10.1002/advs.202201806

**Published:** 2022-09-08

**Authors:** Robert Winkler, Alexander Zintler, Stefan Petzold, Eszter Piros, Nico Kaiser, Tobias Vogel, Déspina Nasiou, Keith P. McKenna, Leopoldo Molina‐Luna, Lambert Alff

**Affiliations:** ^1^ Advanced Thin Film Technology Division Institute of Materials Science Technical University of Darmstadt Alarich‐Weiss‐Straße 2 64287 Darmstadt Germany; ^2^ Advanced Electron Microscopy Division Institute of Materials Science Technical University of Darmstadt Alarich‐Weiss‐Straße 2 64287 Darmstadt Germany; ^3^ Department of Physics The University of York York YO10 5DD UK

**Keywords:** first principle calculation, grain boundary atomic structures, hafnium oxide, resistive switching memory, scanning transmission electron microscopy

## Abstract

Resistive random‐access memories are promising candidates for novel computer architectures such as in‐memory computing, multilevel data storage, and neuromorphics. Their working principle is based on electrically stimulated materials changes that allow access to two (digital), multiple (multilevel), or quasi‐continuous (analog) resistive states. However, the stochastic nature of forming and switching the conductive pathway involves complex atomistic defect configurations resulting in considerable variability. This paper reveals that the intricate interplay of 0D and 2D defects can be engineered to achieve reproducible and controlled low‐voltage formation of conducting filaments. The author find that the orientation of grain boundaries in polycrystalline HfO*_x_* is directly related to the required forming voltage of the conducting filaments, unravelling a neglected origin of variability. Based on the realistic atomic structure of grain boundaries obtained from ultra‐high resolution imaging combined with first‐principles calculations including local strain, this paper shows how oxygen vacancy segregation energies and the associated electronic states in the vicinity of the Fermi level govern the formation of conductive pathways in memristive devices. These findings are applicable to non‐amorphous valence change filamentary type memristive device. The results demonstrate that a fundamental atomistic understanding of defect chemistry is pivotal to design memristors as key element of future electronics.

## Graphical abstract

By combining ultra‐high‐resolution experimental and theoretical methods, a direct correlation between the grain boundary atomic and electronic structures with the electroforming behavior of TiN/*m*‐HfO_2_/Pt memristors with defined HfO_2_ textures is established. The paper reveals how the interaction of different defect types governs the formation of conductive pathways in memristors based on filamentary switching in oxides.

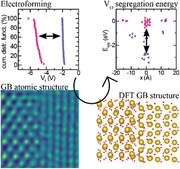

## 1 Introduction

A memristor is based on the controlled (digital or analog)^[^ ^1^, ^2^, ^3^, ^4^ ^]^ change of the resistance of a conductive pathway. In complementary metal oxide semiconductor (CMOS) relevant materials such as Hf—O or Ta—O, the conductive pathways consist of a local enrichment of oxygen vacancies.^[^ ^5^, ^6^, ^7^, ^8^ ^]^ Pre‐existing oxygen vacancies allow for low‐voltage and thickness independent electroforming as only a reshuffle of vacancies is required.^[^ ^9^, ^10^, ^11^, ^12^, ^13^, ^14^ ^]^ However, the oxygen vacancy distribution itself is still a random process associated with forming and operating voltage variability.^[^ ^15^, ^16^, ^17^ ^]^ Defect engineering to reduce the device variability has been investigated in several works using dislocations and nanocomposites.^[^ ^18^, ^19^, ^20^ ^]^ The introduction of threading grain boundaries (GBs) via GB engineering results in highly reproducible low‐voltage electroforming.^[^ ^17^ ^]^ Here, we show that the complete materials picture is disclosed only when taking into account the intricate interplay between point‐defects (oxygen vacancies) and 2D defect planes (grain boundaries). The specific atomic configurations of the grain boundaries including strain effects result in enhanced or suppressed attraction between both types of defects. As a consequence, the selection of the proper GB allows us to create a predefined region of increased oxygen vacancy concentration which is associated with electronic defect states close to the Fermi level. These defect states, in turn, are the nuclei for the soft dielectric breakdown via formation of a defined conducting filament. This insight into the materials defect chemistry suggests new experimental methods of controlling the conducting filament, and thus also serves as a valuable guideline for future memristor designs.^[^ ^21^, ^22^, ^23^, ^24^, ^25^, ^26^, ^27^, ^28^, ^29^, ^30^, ^31^, ^32^, ^33^, ^34^ ^]^

## 2 An Intriguing Observation: Electroforming Voltages Depend on Texture

**Figure 1:**
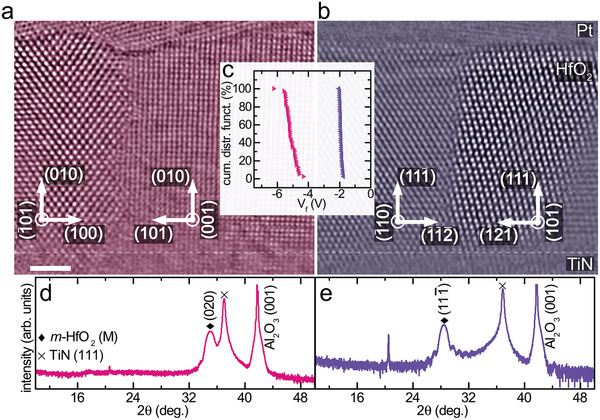
Cross‐sectional high‐angle annular dark‐field (HAADF) scanning transmission electron microscopy (STEM) image of the metal‐insulator‐metal stack with a) (010) and b) ($11\bar{1}$) textured HfO_2_. Scale bar is 2 nm. The TiN‐HfO_2_ interface is indicated by a faint dashed line. Images were filtered by an average background subtraction filter (ABSF), a Butterworth filter and a STEM crosshair filter to reduce image noise.^[^ ^25^, ^26^ ^]^ Titanium nitride (TiN) and hafnium oxide (HfO_2_) have been grown on (001) oriented Al_2_O_3_ by using RMBE. The X‐ray diffraction (XRD) pattern reveals that a change in the growth temperature and rf‐power for HfO_2_ results in d) ($11\bar{1}$), (purple) and e) (020), (pink) when grown at 520 °C, 200 W and 460 °C, 280 W, respectively. c) Devices with (010) textured HfO_2_ (pink) have an increased average forming voltage of $\bar{V_{f}}$= −5.3 V compared to devices with ($11\bar{1}$) HfO_2_ having $\bar{V_{f}}$= −1.9 V as shown by the cumulative distribution function measured from 50 30 × 30 µm^2^ devices. The purple XRD pattern, purple cumulative distribution of *V*_f_ and the purple colored high‐resolution scanning transmission electron microscopy image is adapted from Petzold et al.^[^ ^17^ ^]^

The starting point of this investigation is the discovery of clearly distinct electroforming voltages in memristive model devices with different crystal orientation of the HfO_2_ layer. The devices consist of a 50 nm TiN bottom electrode, followed by a 10 nm thin HfO_2_ layer covered with Pt (100 nm) and gold (300 nm) grown on *c*‐cut sapphire. When choosing the appropriate growth conditions either a ($11\bar{1}$) or (010) texture of the HfO_2_ dielectric is achieved (**Figure** **1**d,e and Figure S1, Supporting Information). We have verified the oxygen content by X‐ray photoelectron spectroscopy revealing that the HfO_2_ layers are homogeneously stoichiometric. Electroforming voltages were collected from 50 devices for each texture and reveal a clear correlation to the respective growth direction of HfO_2_. For the (010) texture an average forming voltage of $\bar{V_{f}}$ = −5.3 V was observed as compared to $\bar{V_{f}}$ = −1.9 V for the ($11\bar{1}$) texture (see Figure 1c and Figure S2, Supporting Information). Both devices have a similar leakage current level at +/− 200 mV (see Figure S2a, Supporting Information). In addition to the very distinct quantitative voltage levels, the device‐to‐device variability is considerably reduced for the low voltage devices which are formed already at standard operation voltages (see Figure 1c).

### 2.1 Texture Defines Specific Grain Boundaries

**Figure 2:**
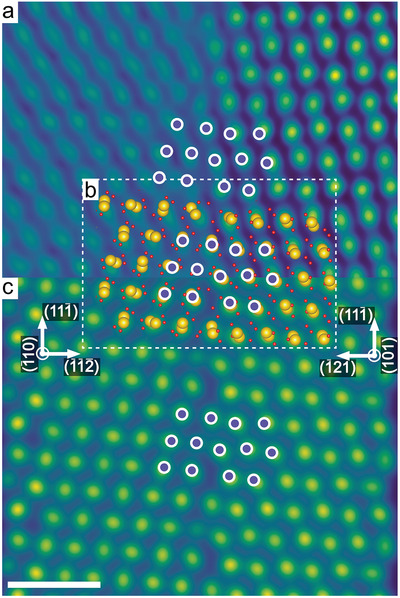
a) Cross‐sectional high‐angle annular dark‐field (HAADF) scanning transmission electron microscopy (STEM) images of a grain boundary in ($11\bar{1}$) textured HfO_2_. The image was used as basis to simulate the b) DFT relaxed atomic structure model which were then used to simulate the HAADF‐STEM image (c). Periodically occurring structural units at the grain boundaries are indicated by purple circles. Scale bar is 1 nm. Experimental HAADF‐STEM images were filtered by an average background subtraction filter (ABSF) and a Butterworth filter to reduce image noise.^[^ ^25^, ^26^ ^]^ All STEM images are colored with the GEM‐16 LUT to improve visibility of atomic columns.

**Figure 3:**
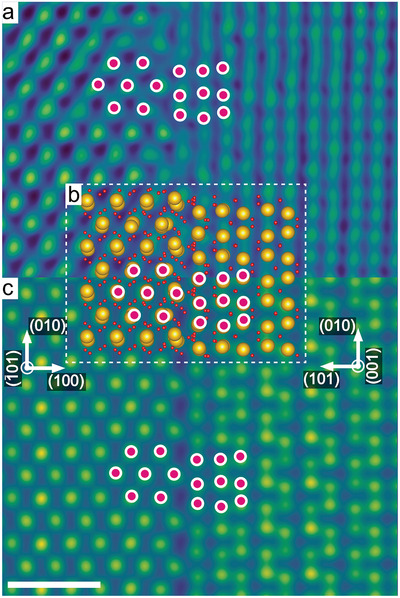
a) Cross‐sectional high‐angle annular dark‐field (HAADF) scanning transmission electron microscopy (STEM) images of a grain boundary in (020) textured HfO_2_. The image was used as basis to simulate the b) DFT relaxed atomic structure model which was then used to simulate the HAADF‐STEM image (c). Periodically occurring structural units at the grain boundaries are indicated by pink circles. Scale bar is 1 nm. Experimental HAADF‐STEM images were filtered by an average background subtraction filter (ABSF) and a Butterworth filter to reduce image noise.^[^ ^25^, ^26^ ^]^ All STEM images are colored with the GEM‐16 LUT to improve visibility of atomic columns.

Transmission electron microscopy revealed that the two types of textured HfO_2_ layers are fully threaded by grain boundaries belonging to well‐defined crystallographic equivalent sets of orientations (see Figure 1a,b). Such grain boundaries display characteristic periodic structure units that repeat themselves along the grain boundary plane (see purple and pink circles in **Figures** **2**a and **3**a). So far, grain boundaries have been discussed as a source of variability.^[^ ^27^ ^]^ Here we show that a smart use of grain boundaries may lead to the opposite, reduced, and more uniformly distributed forming voltage at negative bias. It is plausible to assume that grain boundaries in general provide a predefined pathway for filament formation.^[^ ^27^ ^]^ What is unexpected, however, is that there is a pronounced correlation of crystallographic directions and forming voltages, the latter ones being related to the electronic structure of the grain boundary. In the next step, therefore, we have used high‐resolution imaging of atomic sites to model the real nanostructure of both grain boundary types using density functional theory (DFT). The DFT relaxed atomic structure for the ($11\bar{1}$) textured HfO_2_ (see Figure 2b) is composed of one grain terminated by ($11\bar{2}$) and the other by ($\bar{1}21$). For (020) HfO_2_ (Figure 3b), grains terminate at (100) and (101). The complete unit cells of these structures are shown in Figure S3, Supporting Information. The same periodically occurring structural units are again marked by purple and pink circles. The DFT calculation of the ($\bar{112}$)|($\bar{1}21$) grain boundary revealed two possible stable structures differing only by a rigid translation of one grain with respect to each other (Figure S4, Supporting Information). To validate if these models replicate the real complex atomic structures, multislice‐based electron microscopy image simulations were performed (Figures 2c and 3c). The results match with high accuracy the atomically resolved HAADF‐STEM data. This result shows that imaging of defect processes at grain boundaries can be obtained not only on well‐defined bicrystals,^[^ ^28^, ^29^ ^]^ but can be extended to realistic CMOS materials.

It is important to note that for ($11\bar{1}$) textured HfO_2_, construction of the ($\bar{112}$)|($\bar{1}21$) GB was only possible by applying significant strain parallel to the grain boundary. In contrast, the construction of the (100)|(101) grain boundary for (020) HfO_2_ requires no strain most likely due to the semi‐coherent nature of this grain boundary.^[^ ^30^ ^]^ Strain effects by lattice mismatch or dopants are being routinely used in semiconductor technology to modify electronic properties and transistor performance.^[^ ^31^ ^]^ Here we show that texture transfer induced strain in coherent grain boundaries have a strong effect on electronic properties in memristive devices.

### 2.2 Grain Boundary Influence on Oxygen Segregation Energies and Electronic Structure

**Figure 4:**
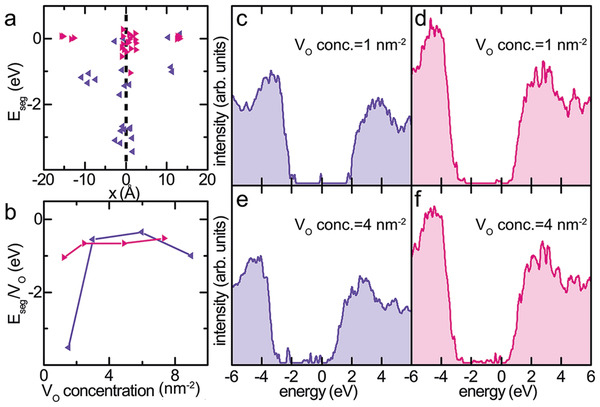
Oxygen vacancy segregation energies were calculated from the DFT relaxed atomic structure models as a function of a) distance from the grain boundary (marked by dotted line) and b) oxygen vacancy concentration for ($11\bar{1}$), (purple) and (020), and (pink) textured HfO_2_. The density of states (DOS) at grain boundaries for c,e) ($11\bar{1}$) and d,f) (020) HfO_2_ were calculated using the projector augmented wave (PAW) method and are shown as a function of oxygen vacancy concentration (*V*_O_ conc.).

Having properly modelled the grain boundary defect nanostructures of realistic memristive model devices gives us the possibility to investigate neutral oxygen vacancy (*V*_O_) interactions with GBs by first principles calculations. Note that we are not using a model grain boundary of higher symmetric (cubic) HfO_2_ but are taking the real atomic positions of the grain boundary within the monoclinic phase. The variation of segregation energy *E*_seg_ in the vicinity of the GBs (**Figure** **4**a) shows that oxygen vacancy segregation is more favorable at the ($\bar{112}$)|($\bar{1}21$) GB (purple) than at the (100)|(101) GB (pink). However, as the concentration of vacancies increases beyond 2 nm^–2^ the average segregation energy per vacancy (*E*_seg_ */V*_O_) for the two GBs becomes comparable (Figure 4b). The electronic properties investigated by the density of states (DOS) projected onto the GB regions (Figure 4c) as a function of *V*_O_ concentration reveal a larger band gap for the ($\bar{112}$)|($\bar{1}21$) GB (purple) than the (100)|(101) GB (pink). The magnitude of the band gaps is comparable to previous reports^[^ ^32^, ^33^ ^]^ and in the presence of *V*_O_ the bandgaps are significantly smaller as compared to bulk *m*‐HfO_2_.^[^ ^34^, ^35^ ^]^ For low *V*_O_ concentration (below 2 nm^‐2^) the DOS of both GBs show a deep and narrow gap state associated with the vacancies.^[^ ^34^ ^]^

For the ($\bar{112}$)|($\bar{1}21$) GB, the gap state is positioned mid‐gap, while for the (100)|(101) GB the gap state is positioned closer to the conduction band. With increase in *V*_O_ concentration the DOS shows the formation of a conductive sub‐band as suggested in literature.^[^ ^33^, ^35^ ^]^ DOS for *V*_O_ concentration equal to 0, 2, and 6 nm^–2^ are shown in Figure S5, Supporting Information.

The electronic properties investigated by the DOS from the DFT relaxed structures of the ($\bar{112}$)|($\bar{1}21$) GB and the (100)|(101) GB were comparable and the introduction of oxygen vacancies resulted in a similar change of the intermediate bandgap states of the DOS. The formed conductive sub‐band for *V*_O_ concentration above 2 nm^–2^ is a percolation path for the current and deteriorates the insulating properties of HfO_2_. The oxygen vacancy concentration can be driven even to a state where the insulator transitions to a semiconducting or even metallic state.^[^ ^35^ ^]^ The presence of preexisting oxygen vacancies is known to reduce the required forming voltages for a conductive filament in HfO_2−_ *_x_*.^[^ ^12^ ^]^ For the here discussed samples, the stoichiometry of the HfO_2_ matrix is close to the ideal 1:2. It can, therefore, be excluded that any anisotropy in oxygen vacancy distribution inside the nanocrystals is the reason for the difference in forming voltages. Therefore, the *V*_O_ concentration below 2 nm^–2^ is representative for a pristine non‐formed resistive switching device, which is insulating. Our results show that at low vacancy concentrations the *V*_O_ segregation energy in ($\bar{112}$)|($\bar{1}21$) GBs (in comparison to the (100)|(101) GBs) are more negative. The ($\bar{112}$)|($\bar{1}21$) GB therefore acts as a sink for oxygen vacancies, thereby, driving the surrounding matrix closer to perfect stoichiometry by the depletion of vacancies, while the grain boundary plane itself is enriched in vacancies. As a consequence, this type of grain boundary is the perfect percolation path for electron transport initiating the formation of a conducting filament.

Due to the monoclinic structure of the HfO_2_ thin films, imaging a grain boundary with both grains being atomically resolved was highly challenging. Moreover, finding a high resolved GB did not guarantee that the construction of a DFT relaxed supercell was possible due to the non‐commensurate nature of adjacent monoclinic grains. Therefore, only one GB of each HfO_2_ texture is shown. However, the textured growth of the ($11\bar{1}$) and (020) HfO_2_ thin films results in six allowed in‐plane directions each (Figure S6a,b, Supporting Information) limiting the possible orientations and resultant GB types. According to Figure S6c,d, Supporting Information, the majority of GBs in (020) textured HfO_2_ are therefore composed of grains terminated by low‐index orientations (e.g., (100)), contrary to GBs in ($11\bar{1}$) HfO_2_ which have high‐indexed terminated grains. Construction of the GB with high‐indexed terminated grains (($\bar{112}$)|($\bar{1}21$) GB) required application of strain. In the near future, we plan to probe local stress fields of GBs with high‐ and low‐indexed terminating grains to investigate the effect on local ionic mobility.^[^ ^36^, ^37^, ^38^, ^39^, ^40^, ^41^ ^]^

## 3 Conclusion

Our combined experimental and ab initio study reveals that an intricate interplay of defects with different dimensionality plays the key role in predefining the formation path of the conducting filament. The controlled induction of a specific grain boundary type by texture transfer is a promising way to overcome present limitations set by the variability of forming voltages and is likely to be favorable for improved device endurance and cycle stability. For the sake of this study we have used MBE growth parameters outside the CMOS temperature budget. However, the favorable combination of a (111) oriented TiN electrode with a (11‐1) oriented HfO_2_ functional layer can also be achieved with industrial relevant methods such as atomic layer deposition. The here suggested method of grain boundary and defect engineering is scalable well below 10 nm as the grain size is comparable to the thickness of the dielectric layer.

## 4 Experimental Section

### Sample Preparation

Reactive molecular beam epitaxy (RMBE) was used to grow TiN on *c*‐cut sapphire followed by in situ growing HfO_2_ in a custom designed ultra‐high vacuum chamber (base pressure ≈ 10^‐9^ mbar).^[^ ^42^ ^]^ For the TiN layers, the substrate was heated to 800 °C. Ti from Lesker (4.5 n purity) was evaporated at a rate of 0.3 Å s^−1^ with in situ nitridation by using a radio frequency (rf)‐source with an rf‐power of 350 W and a flow of 0.6 sccm nitrogen (5 n purity). Growth of hafnia layers with defined textures were achieved by using distinct temperatures and rf‐powers. Hf from MaTeck (3 n purity) was evaporated at a rate of 0.7 Å s^−1^ and in situ oxidized by a rf‐source with a flow of 1 sccm oxygen (5 n purity). For the ($11\bar{1}$) textured hafnia, the rf‐source was operated at 200 W and the substrate was heated to 520 °C. The (020) textured hafnia was grown at a substrate temperature of 460 °C with the rf‐source operating at 280 W. The film thickness and surface crystallinity were in situ monitored by using quartz crystal microbalance (QCM) and reflection high energy electron diffraction (RHEED), respectively. To complement the metal–insulator–metal (MIM) structure, first a standard lithography step was carried out to pattern the surface followed by sputtering 100 nm Pt and 300 nm Au with a Quorum sputter cutter. The lift‐off process resulted in 30 × 30 µm^2^ MIM devices.

### X‐Ray Measurements

Before completing the MIM structure, texture quality of the reactive molecular beam epitaxy (RMBE)‐grown TiN and HfO_2_ thin films were examined with X‐ray diffractometry (XRD). A Rigaku SmartLab diffractometer were used in parallel beam geometry with a copper K_*α*_ X‐ray source.

### Electroforming

Initialization of resistive switching for a device under test (DUT) was achieved by a DC‐negative voltage sweep using a Keithley 4200 semiconductor characterization system (SCS). The top electrode of the device under test (DUT) was biased while electrically grounding the bottom electrode. To prevent a hard dielectric breakdown during electroforming, the internal current compliance (CC) of the semiconductor characterization system (SCS) was limited to 10 and 100 µA.

### Electron Microscopy

From the MIM devices, TEM lamellae were prepared by using in situ lift‐out focused ion beam (FIB) microscopy (JEOL JIB‐4600F). Aberration corrected STEM images were acquired with a convergence angle of 36 mrad by using a field‐emission (S)TEM (JEOL ARM‐200F) at an acceleration voltage of 200 kV. Detection angles of the HAADF detector ranged from 90 to 370 mrad. Growth direction and zone axis of the HfO_2_ thin films were identified by using the diffractGUI of CrysTBox developed by Klinger and Jäger^[^ ^43^ ^]^ with the FFTs of individual grains and a reference structure from Christensen and An^[^ ^44^ ^]^ as the material input.

### Density Functional Theory Methodology

Calculations of the structure and electronic properties of *m*‐HfO_2_ GBs were performed using the projector augmented wave (PAW) method as implemented in the Vienna Ab‐initio Simulation Package (VASP).^[^ ^45^, ^46^ ^]^ The approach was the same as that described in the authors’ previous paper which focused on a single GB type observed in ($11\bar{1}$) textured films.^[^ ^17^ ^]^ Here a second GB type observed in (020) textured films was also considered as well as *V*_O_ segregation to both GB models was considered in order to provide insight into the origin of the difference in *V*_F_. The generalized gradient approximation of Perdew, Burke, and Ernzerhof (PBE)^[^ ^47^ ^]^ and standard Hf and soft oxygen projector augmented wave (PAW) potentials was employed. Using a 400 eV plane wave cut‐off and a Monkhorst–Pack k‐point grid of 11 × 11 × 11 lattice constants for bulk *m*‐HfO_2_ within 0.5% of experimental values were obtained.

The GB in the ($11\bar{1}$) textured films could be formed by joining a ($\bar{1}\bar{1}\bar{2}$) terminated *m*‐HfO_2_ grain to a ($\bar{1}$21) terminated grain. To make a commensurate supercell in this case one must apply strain to both grains parallel to the interface (<10%). The grain boundary in the (020) textured films could be formed by joining a (100) terminated *m*‐HfO_2_ grain to a (101) terminated grain. In this case, it was possible to form a commensurate supercell with very little strain (<0.1%). For both GB models, a vacuum gap of 10 Å was included to separate the two free surfaces in the periodic supercell such that only one GB interface was present. This was necessary in this case as the low symmetry of the GB makes it impossible to construct two equivalent GB interfaces in the supercell as is usually possible for higher symmetry GBs.^[^ ^48^, ^49^, ^50^, ^51^ ^]^ To determine the most stable configuration, a gamma‐surface scan of GB translation states was carried out (in steps of ≈1 Å in both directions parallel to the grain boundary).

To assess the tendency of *V*_O_ to segregate to the GBs, vacancy formation energies for all inequivalent oxygen sites in the region between the centers of each grain were calculated. The formation energy was computed relative to half the energy of an oxygen molecule. For the (020) textured GB (which had negligible in‐plane strain applied), the formation energies in the bulk of each grain were very similar and in good agreement with previous calculations of *V*_O_ in bulk *m*‐HfO_2_ (6.57 eV for three‐coordinated sites and 6.64 eV for four‐coordinated sites). Because the ($11\bar{1}$) textured grain boundary had significant strain applied, the absolute formation energies in the bulk of each grain were modified (≈1.2 eV lower in energy). To allow comparison and assess general trends in defect segregation, a segregation energy (*E_seg_*) is defined relative to the bulk formation energy in each GB model. In both models, vacancy segregation was favorable both in the dilute limit and, as the concentration of vacancies segregated to the grain boundaries, was increased. To assess the effect this has on electronic properties, the electronic DOS both for the pristine stoichiometric GB and the GB with increasing concentrations of *V*_O_ were computed.

### Multislice Simulations

To correlate experimental high‐resolution data to the developed supercells from density functional calculations, STEM images were simulated with the software Dr. Probe^[^ ^52^ ^]^ using the multislice method.^[^ ^53^ ^]^ Within the software, the microscope parameters were set to an acceleration voltage of 200 kV, a convergence angle of 25 mrad, a source size of 0.08 nm, and a HAADF detection range of 90 to 370 mrad with all aberrations set to zero. Cell sizes for equidistant slice creation along *z* with applied Debye–Waller factors for the ($\bar{112}$)|($\bar{1}21$) and the (100)|(101) GB were 10.49 × 1.90 × 14.03 nm, 4800 pixel × 864 pixel × 80 slices, 19 200 atoms and 11.77 × 1.03 × 15.87 nm, 5400 pixel × 480 pixel × 180 slices, 13 920 atoms (x × y × z), respectively. Centered calculation windows were set to 5.24 × 0.95 nm, 524 × 95 pixels and 5.89 × 0.52 nm, 589×52 pixels (x × y) with a sampling of 0.01 nm/pix for the ($\bar{112}$)|($\bar{1}21$) and the (100)|(101) GB, respectively. Computational time for a “Scan image” type with a thickness of ≈10 nm (56 and 113 slices, respectively) took ≈2 h 30 min and ≈2 h for the ($\bar{112}$)|($\bar{1}21$) and the (100)|(101) GB, respectively, using 30 threads of an AMD Ryzen 9 3950X CPU overclocked to 4.15 GHz assisted by a GeForce RTX 3070 GPU (128 GB 2666 MHz RAM and 500 GB Samsung SSD 970 EVO Plus).

## Conflict of Interest

The authors declare no conflict of interest.

## Author Contributions

L.A. and S.P. started and suggested the study. L.A., L.M.‐L. supervised and steered the ongoing project. R.W., N.K., S.P., and T.V. were responsible for sample synthesis and electrical characterization. R.W., D.N., and A.Z. performed the TEM measurements and multislice simulations. K.P.M. performed the DFT calculations. L.A., R.W., L.M.‐L., E.P., S.P., and A.Z. wrote the manuscript. All authors discussed the results and provided constructive comments on the manuscript.

## Supporting information

Supporting Information

## Data Availability

The data that support the findings of this study are available from the corresponding author upon reasonable request.
