# Supplementary material for: Controlling the Formation of Conductive Pathways in Memristive Devices
Source: Adv Sci (Weinh). 2022 Sep 8;9(33):2201806. doi: 10.1002/advs.202201806 (PMC9685438; doi:10.1002/advs.202201806)
Supplement: Supplementary file 1 — Supporting Information [file ADVS-9-2201806-s001.pdf]

ADVANCED SCIENCE
Open Access

Supporting Information

for *Adv. Sci.*, DOI 10.1002/advs.202201806

Controlling the Formation of Conductive Pathways in Memristive Devices

Robert Winkler, Alexander Zintler, Stefan Petzold, Eszter Piros, Nico Kaiser, Tobias Vogel, Déspina Nasiou, Keith P. McKenna, Leopoldo Molina-Luna and Lambert Alff**

Supporting Information

Controlling the formation of conductive pathways in memristive devices

Robert Winkler, Alexander Zintler, Stefan Petzold, Eszter Piros, Nico Kaiser, Tobias Vogel, Déspina Nasiou, Keith P. McKenna, Leopoldo Molina-Luna & Lambert Alff

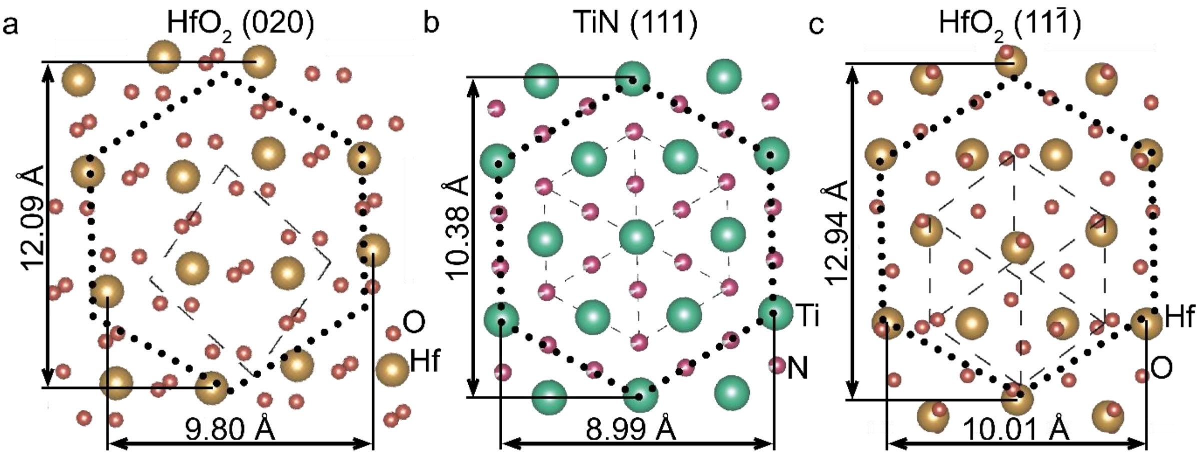

Figure S1. a)-c) To visualize the texture transfer for HfO_2 films grown on TiN, crystal structures of one atomic plane are shown along growth directions for (020), a) and $(11\bar{1})$, c) HfO_2 and (111) TiN, b). The common structure (dotted lines) highlights that the lattice mismatch is smallest between (020) textured HfO_2 and (111) TiN. Unit cells are represented by hatched lines. The structures for TiN and HfO_2 are taken from Christensen & An [1] and Ruh & Corfield [2], respectively and are visualized with VESTA[3].

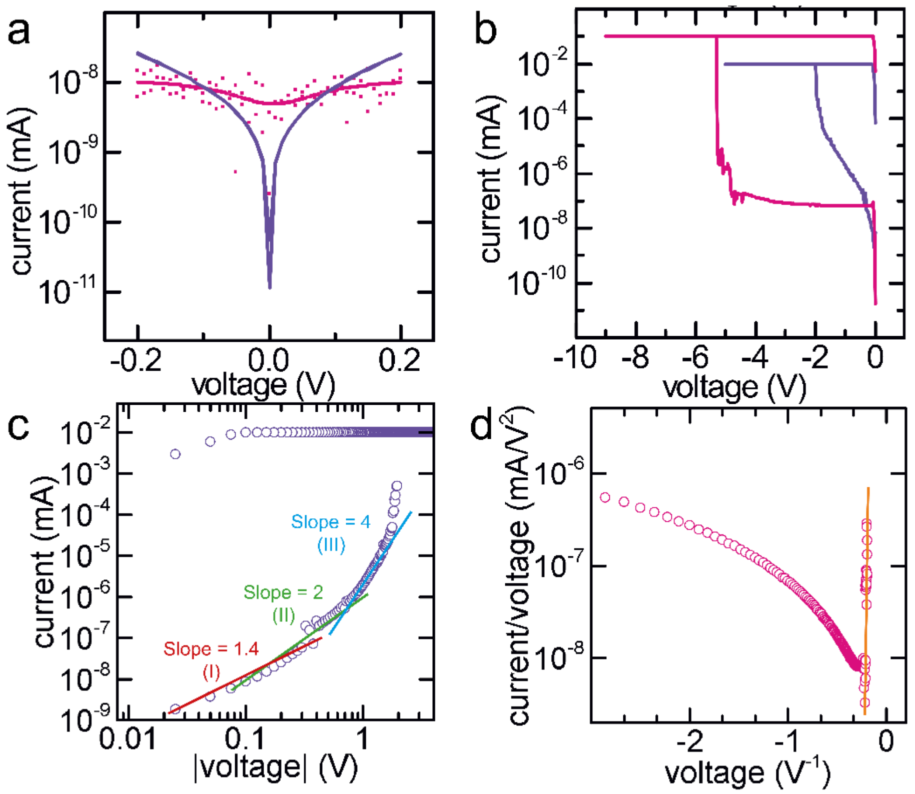

Figure S2. a) Leakage currents of 30x30 µm² TiN/HfO_2/Pt/Au devices are lower for (11$\bar{1}$), (purple) textured HfO_2 compared to a device with (020) HfO_2, (pink). Experimental data of the leakage current for the device with (020) HfO_2 were fitted by a Lorentzian function. b) The required forming voltage is greatly reduced for a device with (11$\bar{1}$) HfO_2 (purple). (c) The initial forming process for the device with (11$\bar{1}$) hafnia is refined to a space-charge-limited conduction (SCLC) mechanism with a trap-filled limit voltage around 1 V. (d) For the device with (020) textured hafnia the conduction mechanism is consistent with Fowler-Nordheim tunneling ($\ln {}^{I}/_{V^2} \sim {}^{1}/_{V}$).

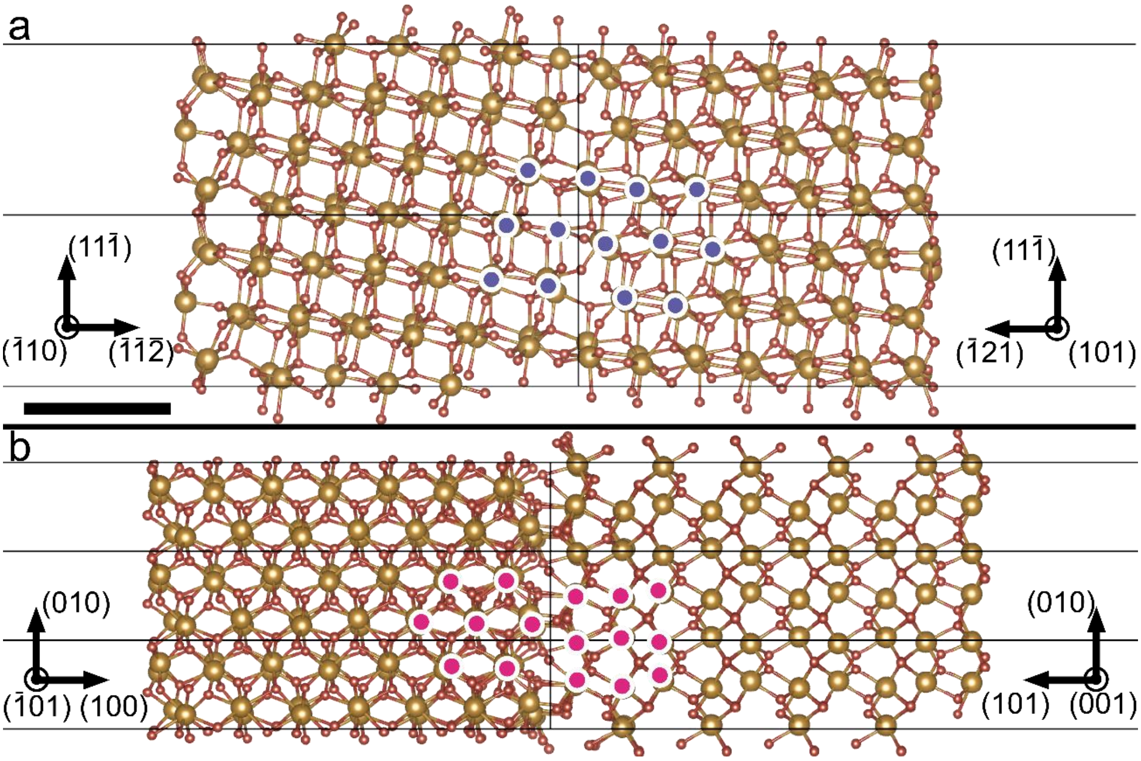

Figure S3. DFT relaxed atomic structures were retrieved from the HAADF-STEM images of grain boundaries (Fig. 2) for a) $(11\bar{1})$ and b) (020) textured HfO_2. The same periodically occurring structural units are marked in purple and pink. Scale bar is 1 nm. The DFT structures are based on a model from Ruh & Corfield[2] and are visualized with VESTA[3].

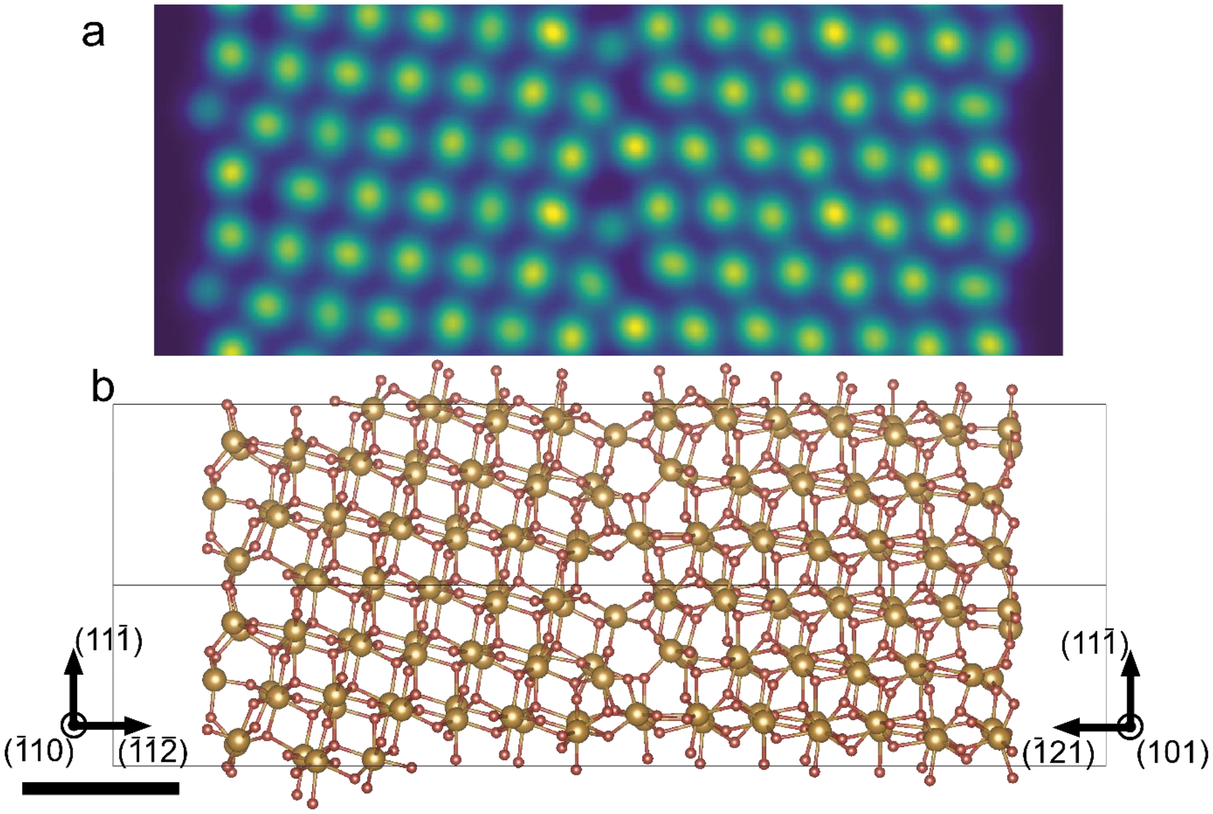

Figure S4. a) The simulated HADDF-STEM images retrieved from b) the second stable DFT structure of (11$\bar{1}$) textured HfO_2. Scale bar is 1 nm. The DFT structures are based on a model from Ruh & Corfield [2] and are visualized with VESTA [3].

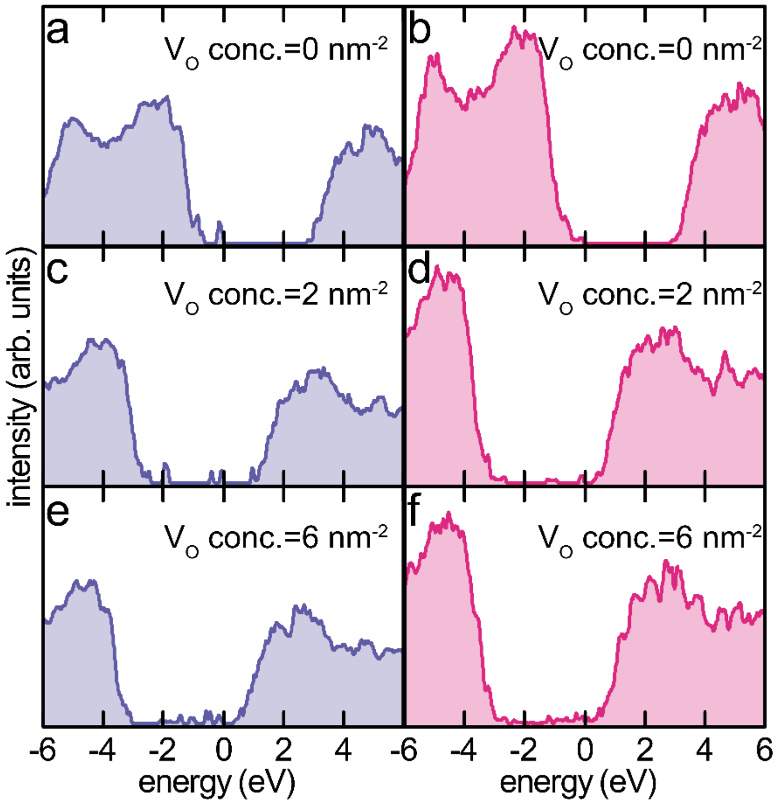

Figure S5. a), b) The density of states (DOS) show no intermediate gap states at an oxygen vacancy (V_O) concentration (conc.) equal to zero. c), d) Multiple intermediate states for a V_O conc. = 2 nm^{-2} and e), f) a conducting sub band at V_0 conc. = 6 nm^{-2} for the $(11\bar{1})$, (purple) and (020), (pink) HfO_2 grain boundaries. The DOS were calculated using the projector augmented wave (PAW) method.

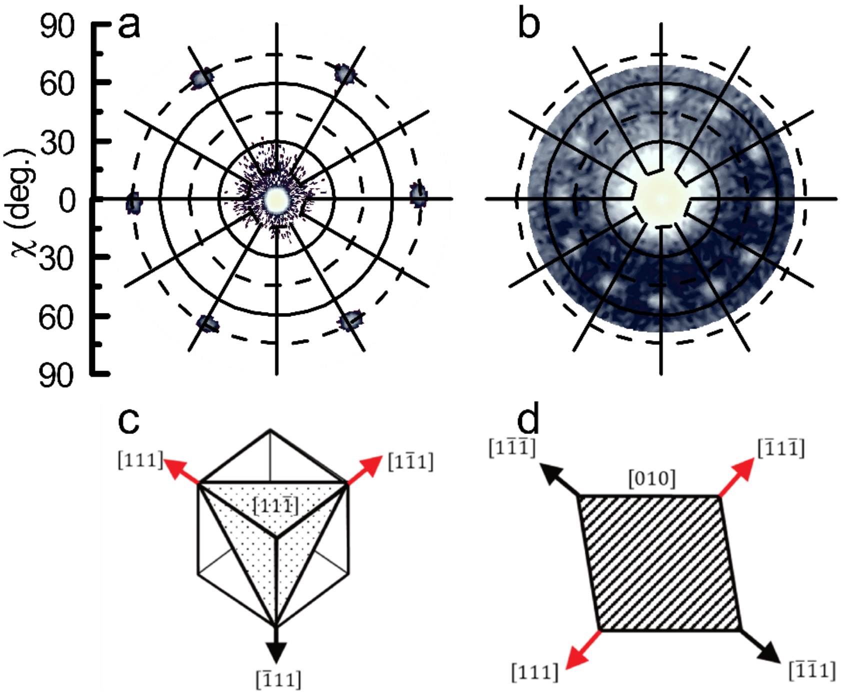

Figure S6. The (11$\bar{1}$) pole figures (PFs) for a) (11$\bar{1}$) and b) (020) textured HfO_2 reveal six and 12 clustered poles, respectively. Due to the monoclinic structure, one defined in-plane orientation should result in one pole for the c) (11$\bar{1}$) and two poles for the d) (020) textured HfO_2 when measuring the (11$\bar{1}$) PF indicated by the black (measurable poles) and red (not measurable poles) arrows. Hence, six defined in-plane orientations are expected rotated in-plane by 60° and 30° for (11$\bar{1}$) and (020) textured HfO_2, respectively.

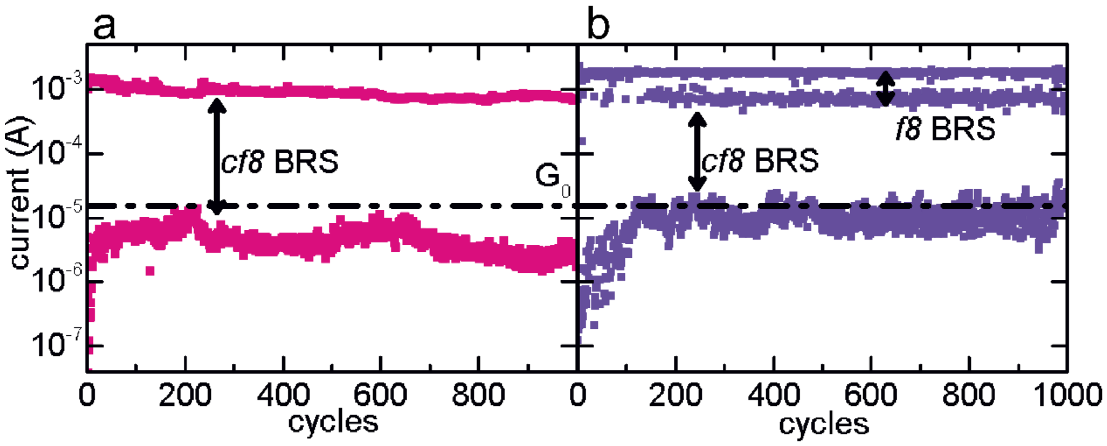

Figure S7. DC endurance behavior trends of devices with (a) (020) and (b) $(11\bar{1})$ textured hafnia for current readout at 200 mV. The device based on (020) textured HfO_2 shows bipolar resistive switching in the so-called counter figure eight (*cf8*) mode. In the device based on $(11\bar{1})$ textured HfO_2 the high resistance value locks to the conductance quantum G_0 at $(12.9\ k\Omega)^{-1}$ (dashed line) after several cycles. The low resistive state shows two well-defined values attributed to *cf8* and figure 8 (*f8*) switching. These two states occur when switching takes place at both electrodes (see S. U. Sharath *et al*., Adv. Funct. Mater. **2017**, 27, 1700432).

References

[1] A. Christensen, C. An, The Temperature Factor Parameters of Some Transition Metal Carbides and Nitrides by Single Crystal X-ray and Neutron Diffraction. **1978**.

[2] R. Ruh, P. W. R. Corfield, Journal of the American Ceramic Society **1970**, 53, 126.

[3] K. Momma, F. Izumi, J Appl Cryst **2011**, 44, 1272.
